# School students' burdens and resources after 2 years of COVID-19 in Austria: a qualitative study using content analysis

**DOI:** 10.3389/fpubh.2024.1327999

**Published:** 2024-02-09

**Authors:** Marlies Sobotka, Thomas Kern, Katja Haider, Rachel Dale, Veronika Wöhrer, Christoph Pieh, Thomas Probst, Elke Humer, Andrea Jesser

**Affiliations:** ^1^Department for Psychosomatic Medicine and Psychotherapy, University for Continuing Education Krems, Krems an der Donau, Austria; ^2^Department of Education, University of Vienna, Vienna, Austria; ^3^Division of Psychotherapy, Department of Psychology, Paris Lodron University Salzburg, Salzburg, Austria; ^4^Faculty of Psychotherapy Science, Sigmund Freud University Vienna, Vienna, Austria

**Keywords:** adolescents, COVID-19, mental health, qualitative content analysis, resources, burdens, school students

## Abstract

**Introduction:**

The mental health of young people has been severely affected by the COVID-19 pandemic and the measures associated with it. Mental health symptoms increased across various domains during the pandemic and subjective wellbeing decreased.

**Methods:**

This study examined the burdens and resources of Austrian school students (*M* = 16.63 years old) in the third year of the pandemic and compared them descriptively with the burdens and resources identified in a comparative study in 2021. A cross-sectional online survey with open-ended questions from April to May 2022 provided data that was analyzed using qualitative content analysis. A sub-sample of *N* = 214 was selected from the larger sample. This sub-sample is representative for the Austrian population aged 15–19 in terms of gender and migrant background.

**Results:**

Analysis of the open-ended questions showed that concerns about the pandemic and the burdens of the measures were no longer on young people's minds at the time of the survey in 2022. On the other hand, stress at school was increasing and the opening of schools and the resumption of face-to-face teaching were challenging for respondents. While resuming social contacts and leisure activities was mentioned as a resource by many respondents, some also expressed a desire for more time off and a retreat into coping strategies such as sleeping more or avoiding problems.

**Discussion:**

Our findings suggest that there is a need for low-threshold support from teachers and parents to help students catch up on missed lessons and to empathize with the mas they make the transition back to “old normal”.

## 1 Introduction

The phase of adolescence poses a range of challenges in an individual's developmental journey. During this period, adolescents must navigate various developmental tasks to evolve into independent and responsible adults (1). The COVID-19 pandemic introduced a multitude of additional disruptions that significantly impacted the lives of young individuals. Numerous studies have demonstrated that adolescents faced a range of challenges during this time that affected their development and wellbeing (2–7).

Study findings from a relatively early phase of the pandemic already indicate significant negative impacts on the quality of life and mental wellbeing of adolescents and students, increasing the risk of mental health problems (8). A large German study revealed that 70.7% of children and adolescents felt significantly burdened by the pandemic and its associated changes, with 64.4% finding homeschooling particularly stressful and 82.8% expressing dissatisfaction with the lack of contact with friends (9–12). While there was an initial deterioration in health-related quality of life and mental health at the onset of the COVID-19 pandemic in 2020, improvements were noted after 2 years. Nevertheless, these improvements had not yet returned to levels seen before the pandemic (13–15).

While quantitative data allow us to assess the impact of the pandemic on mental health over the course of the pandemic, qualitative research can help us to better understand the different subjective experiences that lead to increased mental distress, or to get a more detailed picture of the resources and coping strategies of young people in different life situations.

Qualitative studies from the onset of the pandemic found that young people were particularly burdened (16–20) by academic challenges such as coping with schoolwork, maintaining academic motivation and adapting to distance learning (21). The limitations on social life and the obligation for physical distancing posed another significant challenge. Young people experienced a loss of friendships, social skills, and important life moments such as graduation ceremonies, and birthdays (22–24). In this overall context, they reported experiencing psychological distress, including feelings of boredom, monotony, depression, anxiety, and loneliness, as well as physical burdens, such as headaches and muscle pain (23). Other reports also highlighted young people's loss of autonomy due to government restrictions and their anguish related to death and loss (25).

On the other hand, research findings indicated that for some young people, the early stages of the pandemic also provided opportunities for relief, growth and development. During the lockdowns, adolescents had time to reflect on themselves, to strengthen family ties (25, 26) to identify new and feasible ways of self-care (27) to activate creative resources (28) or to pursue hobbies for which they otherwise lacked time (29). Technology enabled them to progress in school and stay in touch with friends and peers (29). Additionally, for some young people, the temporary switch to distance learning provided relief from academic pressure, difficulties with teachers, and bullying (30).

As the pandemic continued, the exhausting impact of prolonged exposure to pandemic-related burdens became evident in qualitative accounts as well. In 2021 study, Lukoševičiute-Barauskiene reported that young people's narratives were dominated by negative depictions of the pandemic's effects (31). Not being able to see friends, classmates and relatives for a long time was experienced as increasingly difficult and virtual interactions could not replace face-to-face contact in the long term (32, 33). Young people described the feeling of having irretrievably missed out on something in life (33). Distance learning and home confinement caused boredom and despair (33). According to Widnall et al. (30), the ongoing uncertainty and frequent changes in social distancing rules, along with last-minute announcements of school closures and the return to distance learning, were considered burdensome by students in their study from 2021. Bryce and Fraser observed that students found it more challenging to maintain motivation during distance learning (34). Furthermore, distance learning was perceived as less effective, and students expressed concerns about their learning outcomes (30, 31, 33), as well as their academic and career prospects (33, 35, 36). At the time of submission, no publications referring to more recent data were found.

The situation in Austria aligns with the international context. Various studies demonstrated that the mental health of the Austrian population progressively declined since the onset of the pandemic (37–40). Adolescents were particularly affected by the pandemic's repercussions. A cross-sectional study conducted by Pieh et al. in February 2021 among 3052 school students aged 14–20 revealed a significant increase in depressive symptoms, anxiety symptoms, insomnia and disordered eating compared to pre-pandemic data. About one third of respondents reported suicidal thoughts (18). In comparison to the last Health Behavior in School-Aged Children study conducted in 2018 (41), both psychological wellbeing and life satisfaction declined (18). Another Austrian online survey of children and adolescents aged 6 to 18 conducted in spring 2021 revealed a high level of pandemic-related anxiety, a concerning lack of positive outlook during the ongoing pandemic, and an increase in feelings of anger, loneliness, and sadness (42). In spring 2022, Kaltschik et al. conducted a repeated cross-sectional study with 616 students aged between 14 and 20 which revealed that the mental health of Austrian adolescents declined further despite a major easing of COVID-19 restrictions in the third year of the pandemic (43).

Qualitative findings have provided comprehensive insights into elevated levels of mental health stress. In addition to gathering quantitative data on symptom burden, Pieh et al. (18) also collected qualitative data, presenting a nuanced picture of the challenges and resources faced by young people in February 2021, 1 year after the onset of the pandemic. The study comprised two open-ended questions: “What is currently causing you the most concern?” and “What is currently providing you with the most support?”. From the total sample of 3052 adolescents, a representative sample (*N* = 214) with regard to gender and migration background was randomly selected. Qualitative content analysis revealed several areas of concern, including worries related to school, concerns about COVID-19 restrictions, self-related concerns (such as worries about the future, feelings of hopelessness and negative thoughts), and family and interpersonal problems (35). Important sources of support for adolescents included social contacts, leisure activities, attitudes and skills (such as practicing self-reflection, taking an active role in dealing with their problems, thinking positively, having confidence in themselves, meditating, pursuing their goals or laughing), distracting themselves by playing video games alone or with friends online, and escapism (i.e., drinking alcoholic beverages, sleeping, using drugs). Of concern was the proportion of young people who reported maladaptive coping strategies and limited willingness to seek professional help (35).

As the mental health of young people has changed considerably over the course of the pandemic, it is important to document the current situation through ongoing research and to make recommendations on how to support young people. By repeating the qualitative content analysis with data from 616 14–18-year-old Austrian students collected in spring 2022 [for quantitative results see (37)], this study aims to show how young people's burdens and resources have changed over the course of a year.

## 2 Materials and methods

### 2.1 Research design

From April to May 2022, we conducted an online cross-sectional survey among adolescents aged 14 to 20. This study presented in this paper is part of a larger investigation of adolescent mental health following the last COVID-19 lockdown in Austria and the gradual easing of government restrictions. We obtained approval from the Ethics Committee and the Data Protection Officer of the University for Continuing Education Krems (Protocol code: EK GZ41/2018-2021). The study was conducted following the Declaration of Helsinki.

The study was conducted using Research Electronic Data Capture (REDCap) (44, 45), hosted on servers of the University for Continuing Education Krems, Austria. Standardized, validated quantitative questionnaires were used to screen respondents for the presence of mental health problems. To allow respondents to report in their own words without predetermined answers, the survey included two open-ended questions for qualitative analysis: What is currently causing you the most concern? (Question 1) What is currently providing you with the most support? (Question 2) The original questions and responses were formulated in German. Responses ranged from single-word answers to full paragraphs. The quantitative results have already been published (43); in this paper, we compare the results of the qualitative analysis with the results of the 2021 analysis (35) to assess changes in perceived burdens and resources, and to get a picture of young people's experiences at the end of the pandemic.

### 2.2 COVID-19 measures in Austria before and during the survey

In Austria, the first COVID-19 case was reported on February 25, 2020. Three weeks later, the first nationwide lockdown was implemented. Schools, universities, public sports facilities, gardens, gastronomy, and a large portion of commerce were closed. Furthermore, extensive restrictions on movement were imposed. After initial relaxations from mid-April 2020, schools were gradually reopened from early May 2020. The re-opening process was accompanied by extensive safety measures, such as adhering to distancing rules, mandatory mask-wearing, COVID-19 antigen tests for students and teachers, and the implementation of alternating timetable arrangements. A sharp increase in infections in the fall of 2020 led to school closures again from mid-November to December 7, 2020, and subsequently after the Christmas holidays until February 7, 2021 (date of first survey).

Starting from March 2021, Austria faced a third wave of COVID-19. The eastern provinces saw a strict lockdown, while the rest of the country implemented partial lockdown measures, including nighttime curfews and closures in the gastronomy sector. During the summer months, the situation eased. In the autumn, however, COVID-19 infections increased. Another nationwide lockdown was enforced in November 2021, leading to the closure of commerce, gastronomy, and leisure activities. Full-day curfews were imposed, though schools remained open with absences being excused and even recommended. From February 2022 onwards, there were initial relaxations in the school sector and a gradual return to normal operations (46). The organization of school events and excursions became possible again. Furthermore, compulsory attendance at schools was reinstated, and starting from April 25, 2022, the mask mandate was lifted in all school classes (47) (date of second survey).

### 2.3 Sampling frame

A cross-sectional online survey was conducted via REDCap (Research Electronic Data Capture), hosted on the servers of the University for Continuing Education Krems. The survey enrollment period was from 26th April to 24th May 2022. The recruitment strategy involved online convenience sampling, with school representatives being contacted and asked to share an invitation containing the link to the online survey on various social media platforms for students. To be eligible for participation, individuals had do meet the following criteria: (a) age between 14 and 20 years, (b) attendance at an Austrian school, and (c) provision of electronic informed consent. Subsequently, participants completed a survey that included demographic information and mental health questionnaires. Only respondents who provided data on all outcome variables were considered in further analysis.

A total of 616 individuals participated in the survey, with an average age of 16.63 years (standard deviation = 1.49). Among participants, 77.4% identified as female, 19.8% as male, and 2.8% identified as non-binary. Additionally, 18.3% indicated having a migration background. Of the 616 respondents, 593 individuals (96.3%) answered at least one of the open-ended questions, while 576 individuals (93.5%) answered both questions.

For the qualitative analysis in the current paper the sample was taken from this participant pool, with some further inclusion criteria. Dale et al. (48) demonstrated that vocational school students have worse mental health compared to higher secondary school students. For this reason, individuals who reported attending a vocational school, not attending any school, or engaging in other activities (i.e., trainings at the Labor Market Service) were excluded. The focus was thus on adolescents attending their last year of compulsory school or some form of secondary school. Furthermore, only participants who had answered both open-ended questions were included in the sample, regardless of the length of their responses. From those who met the above inclusion criteria, we randomly drew a sample, with the restriction that it be representative for the Austrian population aged 15–19 in terms of gender and migration background based on youth data from Statistics Austria (49). A minimum of 30 individuals per category for gender (female/male) and migration background (yes/no) was set as a criterion. These variables were chosen as previous studies have indicated their significant influence on the mental health of adolescents (50–52). The final sample comprised 214 participants (mean age: 16.56 ± 1.26 years; 50% female, 50% male; 28% migration background).

### 2.4 Data analysis

Data derived from open-ended questions were subjected to a combination of conventional and directed qualitative content analysis (53). Qualitative content analysis is a technique that allows data material to be structured in terms of its main content. The systematic and structured approach allows large amounts of data to be analyzed efficiently. Furthermore, qualitative content analysis is particularly suited for material that lacks context, such as when examining open-ended survey questions. In the conventional approach to qualitative content analysis, researchers approach the material with an open mind. They immerse themselves in the material and form concepts from it. These concepts are then further abstracted into higher-level categories at varying levels. In our study, we intended to approach the material with this openness, but at the same time we wanted to check whether comparability with the results of an earlier investigation using the same study design was possible. To achieve this, we utilized a combination of the conventional and the directive approach. In the directive approach, theoretical assumptions and relevant research findings form the basis of the category system. In our research, we used the coding scheme developed in the previous study conducted in February 2021 as the starting point for the analysis (Supplementary Tables S1, S2). Using Atlas.ti (54) coder 1 coded all data with the deductive coding scheme. At the same time, she inductively developed new categories from the data. Some categories from 2021 were no longer relevant in 2022, for example the subcategories of the category “restrictions” or the subcategory “missing out on something”. On the other hand, new subcategories emerged from the data such as “lack of time” or “making decisions”. These changes in the categories are the result of changing conditions in young people's lives.

Subsequently, coder 2 coded the entire data set again using the extended coding scheme. For this step of the coding process, the assignments of coder 1 were deleted. To enhance the reliability of the coding process and ensure that the two coders were using the categories consistently, their coding was then compared (55). We assessed the agreement between coders by calculating inter-coder reliability scores (56). Discrepancies between coders were discussed individually to ensure that the codes accurately reflect the meaning of the data and are not influenced by individual coder bias (57).

The percentage agreement for “Concerns” was 64.2%, the inter-coder agreement, calculated using Krippendorff's alpha (cαbinary), was 0.948. The percentage agreement for “Support” was 74.3%. The inter-coder agreement for “Support”, calculated using Krippendorff's alpha (cαbinary), was 0.925.

Coder 1 then solved minor coding conflicts by herself. For example, coding rules were sometimes disregarded by coder 2. In another case, categories were named so similarly that coder 2 selected the wrong category from the drop-down menu. The remaining coding conflicts were discussed and solved together by discussing the meaning of each text passage that was allocated differently by the two coders. Overall, the analysis of “Concerns” resulted in a total of 9 categories and 18 subcategories. The analysis of “Support” led to 14 categories and 27 subcategories. The detailed category systems including the frequency of allocated text passages are provided in the Supplementary Tables S3, S4.

## 3 Results

This section compares the results of the qualitative content analysis from 2021, as reported in Jesser et al. (35), with those of 2022, highlighting the areas where changes are evident.

### 3.1 Comparison of sources of concern in February 2021 and April–May 2022

**Figure 1:**
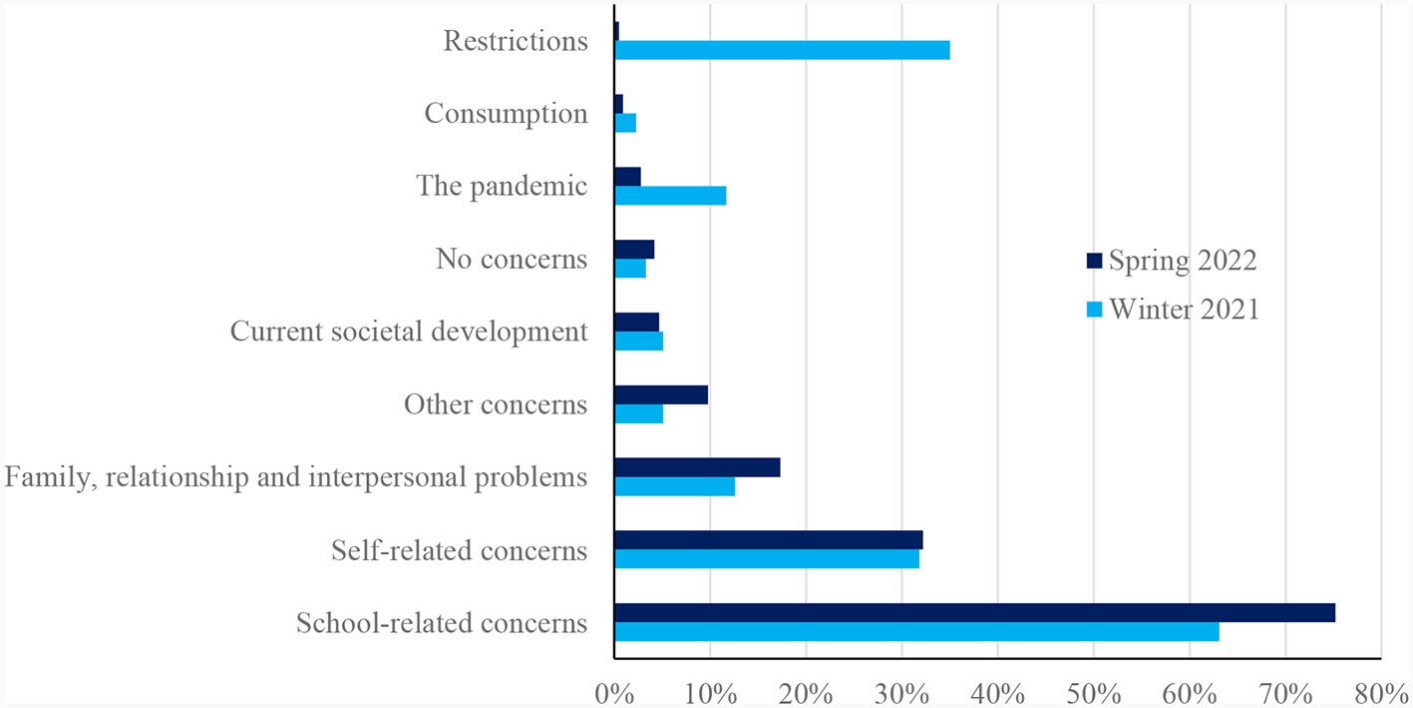
Comparison of the survey results from February 2021 and April to May 2022 regarding the question: “What currently gives you the most cause for concern?”

In the following section, statements about important sources of concern reported by the participants during the survey period from April to May 2022 are compared to the concerns reported in February 2021. Figure 1 compares the main categories in response to the question “What is currently causing you the most concern?” from February 2021 and April to May 2022.

#### 3.1.1 School-related concerns

Although school-related concerns were the most frequently mentioned main category at both time points, this category was mentioned more frequently in 2022 (75.2%) than in 2021 (63.1%). The focus of the answers also shifted, with more reports related to stress at school in 2022 (64.0%) vs. 2021 (43.5%). In this category, we allocated accounts addressing struggles with demands for performance, worries about grades, difficulties in specific subjects and mentions of the workload of homework, of assignments, tests and examinations. The young people's statements in 2022 show their concern that they have missed out on a lot due to the long period of distance learning and that they now have to catch up on their own. For instance, respondent 210 expressed concern about “*exam stress, as much has been lost in the past few years that needs to be caught up on for exams that cover the last 2 years*,” and respondent 25 mentioned “*The fact that I feel abandoned with all the tasks and homework that need to be done.”*

Compared to 2021, when 8.9% of students reported concerns addressing school organization, i.e. the burden of spending hours in front of the computer during distance learning, the strain of interrupted routines due to the constantly changing organization of the school day and alternations between face-to-face teaching and distance learning, issues related to school organization were no longer a significant stressor in 2022 (mentioned by 0.5% students in 2022).

No notable change was found in the subcategories “graduation” (7.9% in 2021 vs. 8.9% in 2022) and “teachers” (2.8% in 2021 vs. 1.9% in 2022). However, some respondents in this category also said that they were nervous about their upcoming school leaving examination because they had been behind in their learning over the last 2 years. For example, respondent 119 criticized, “*I haven't learned anything in school over the past 2 years due to Corona. Online teaching wasn't really teaching, I only learn face-to-face, and now I'm at a loss for the final exams. No idea about anything*.” Students in vocational education felt that they lacked the practical experience necessary to be confident in their upcoming exams.

#### 3.1.2 Pandemic-related concerns

Comparing the data from 2021 and 2022 shows a shift in the second most common category. In February 2021, COVID-19 related restrictions were frequently named as a source of concern (35%). Respondents were burdened by the restrictions of public life and home confinement (13.6%), the lack of activities with friends (9.8%) and the lack of social contacts in general (6.1%). They also mentioned the lack of exercise (2.3%), travel restrictions (1.9%) and quarantine (1.4%) as a burden. In 2022 COVID-19 related restrictions were mentioned by only one student. References to the pandemic, both in general (5.6% in 2021) and regarding future uncertainty (6.1% in 2021), also decreased in 2022 (from 11.7% to 2.8%).

#### 3.1.3 Self-related concerns

The category “self-related concerns” pertains to various aspects of inner experience and sense of self. There were no significant changes in this category, with 31.8% in 2021 and 32.2% in 2022. However, changes are noticeable at the subcategory level.

In 2022, mental health stresses were the most frequently mentioned burden, accounting for 8.4% of responses. In 2021, they were mentioned by only 2.8% of respondents. The text passages in this category specifically mention mental health problems such as depression, eating disorders, panic attacks, and symptoms associated with mental illness, including depressive moods, anxiety, and suicidal thoughts. In 2022, students reported experiencing both new and pre-existing mental health problems. Physical problems on the other hand were mentioned by 2.8% in 2021 vs. 1.4% in 2022.

There were no overall changes in the frequency of the subcategory “negative thought and emotions” (6.1% in 2021 vs. 5.6% in 2022). In 2021, statements in this category expressed young people's self-doubt, anger, and sadness. Such statements were also found in the data material from 2022. However, some of the statements in 2022 express more profound existential doubts and insecurities among young people, as exemplified by the following statement by respondent 104: “*I feel empty and break down almost every evening and cry for half an hour*.” Respondent 142 wrote about the “*overwhelming burden of existence”*, and respondent 141 about “*a constant feeling of being alone, also missing the meaning in life*.”

Due to frequent mentions, “lack of drive” and “loneliness and social isolation” were coded separately in 2021. There were slight changes in both categories in 2022 (3.7% to 2.3% for “lack of drive” and 1.4% to 1.9% for “loneliness and social isolation”). In this category, too, some of the young people's statements point to existential difficulties that were not visible in 2021. Respondents reported lacking even the basic motivation to get up in the morning and do anything at all. They found it difficult to manage daily studying and perceived their presence at school as pointless.

In 2022, a new subcategory was created that did not exist in 2021. “Lack of time” was reported as a burden by 5.1% respondents. They reported experiencing time pressure and a lack of leisure time. They experienced difficulties in managing their time effectively, which made it challenging for them to meet important commitments. They expressed distress when they were unable to accomplish their planned tasks. Conversely, one category no longer appeared in 2022, namely the category “missing out on something”.

Compared to the previous year, concerns about the future decreased in 2022 (10.3% in 2021 vs. 5.6% in 2021). In 2021, young people expressed concerns about future education and careers, making the wrong choices and a lack of future prospects. In 2022, they additionally cited worries about inflation making it difficult to afford living costs and not being able to realize plans. Respondent 111, for example, was burdened by “*the thought of not having a proper plan for my future*.”

“Bullying” was a new category in 2022, mentioned by 0,9% of respondents. The category of “body-related worries” was present in both survey dates (1.4% in 2021 vs. 0.9% in 2022).

#### 3.1.4 Family, relationship and interpersonal problems

The comparison of categories from 2021 and 2022 reveals further changes in the main category “family, relationship and interpersonal problem”, which has seen an increase from 12.6% in 2021 to 17.3% in 2022. In particular, relational problems with others increased (2.8% in 2021 vs. 7% in 2022). In 2022, students mentioned being burdened by a lack of friends, few contact with friends, or having lost friends due to repeated curfews. Regarding family problems (4.2% in 2021 vs. 6.1% in 2022), the young people reported experiencing frequent conflicts at home and a lack of support from their families. Further subcategories at both points of data collection were “relationship troubles” (3.3% in 2021 vs. 3.7% in 2022) and “worrying about others”. In 2022, there were fewer mentions expressing concern for others (2.3% in 2021 vs. 0.5% in 2022).

#### 3.1.5 Further concerns

There was little change in the categories “current societal developments” (5.1% in 20021 vs. 4.7% in 2022) and “no stressors” (3.3% in 2021 vs. 4.2% in 2022). However, the war in Ukraine was introduced as a new topic in 2022. Responses in the category “consumption” decreased slightly (2.3% in 2021 vs. 0.9% in 2022). Responses assigned to the catch-all category “other concerns” increased in 2022 (5.1% in 2021 vs. 9.8% in 2022). Respondents, for example, described concerns about the loss of important people in their lives or worried about their parents' economic situation, such as respondent 34, who mentioned “*little money due to inflation, parents run a restaurant, which is still doing poorly*.” Due to their generality and lacking context (“*Everything*,” “*Many things*”), some responses were difficult to categorize.

### 3.2 Comparison of sources of support in February 2021 and April–May 2022

**Figure 2:**
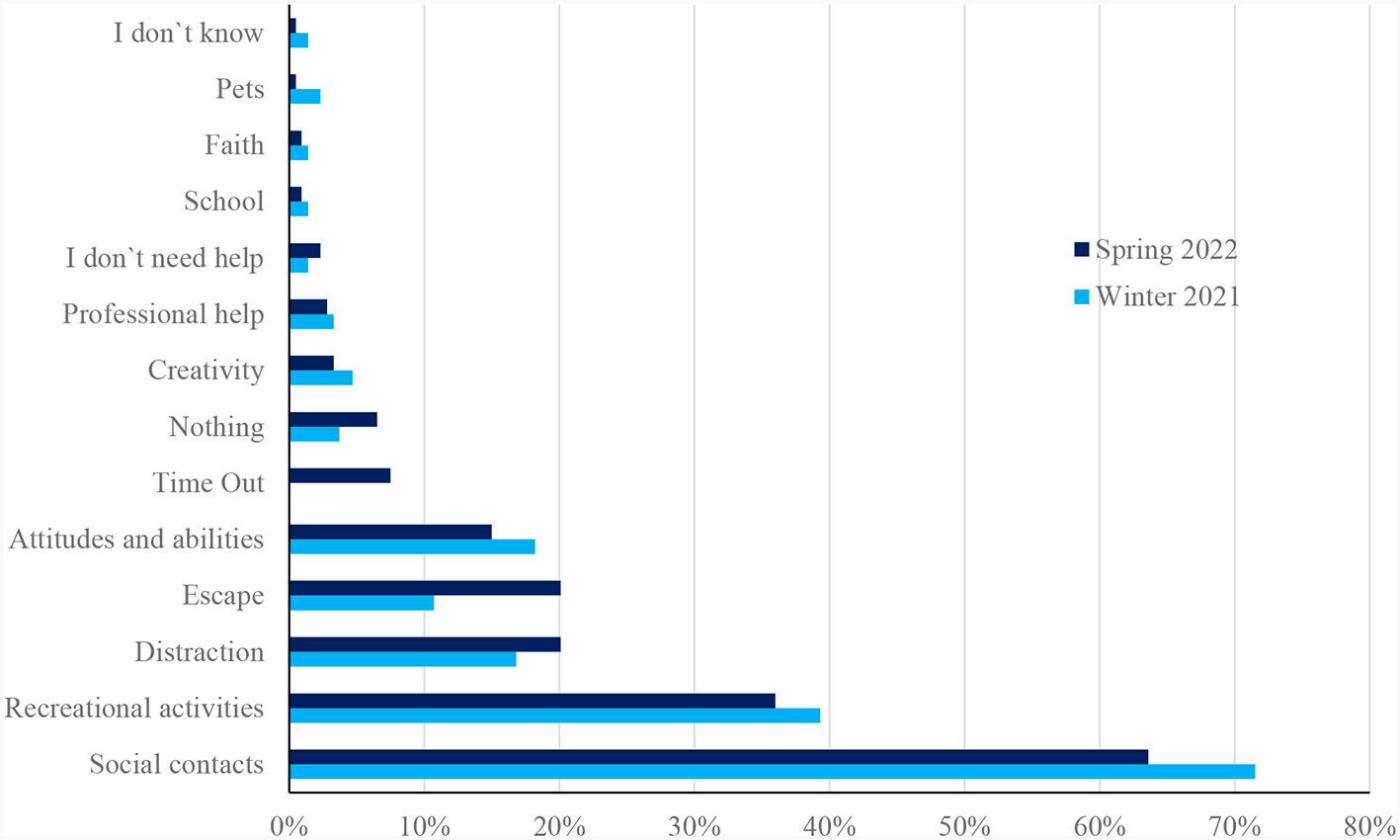
Comparison of the survey results from February 2021 and April to May 2022 regarding the question: “What currently gives you the most cause for support?”

In the following section, statements about important sources of support reported by the participants during the survey period from April to May 2022 are compared to the sources of support reported in February 2021. Figure 2 compares the main categories in response to the question “What is currently providing you with the most support?” from February 2021 and April to May 2022.

#### 3.2.1 Social contacts

When comparing the main sources of support at both time points, the category “social contacts”, mentioned by 71.5% of respondents in 2021 and 63.6% in 2022, stands out. Support from friends or a best friend was mentioned about equally often in 2021 and 2022 (29.9% and 1.9% in 2021 vs. 31.8% and 1.4% in 2022). Most of the responses in 2022 were brief, with students simply stating “friends” or “one friend”. However, some students mentioned that they valued the emotional support provided by their friends, as well as the opportunity to discuss any issues they were having. Additionally, some students appreciated their friends' help with learning assignments and enjoyed participating in activities with them. While the other subcategories remain relatively stable (“talking to someone” with 8.9% of mentions in 2021 and 9.3% in 2022; “partner” with 9.8% vs. 7.5%, “classmates” with 2.8% vs. 1.4%; “other people with 1.4% vs. 2.3%), the category “family support” shows a decrease in mentions (16.8% in 2021 vs. 9.8% in 2021).

#### 3.2.2 Recreational activities

“Recreational activities” were also mentioned less frequently in 2022 than in 2021 (39.3% in 2021 vs. 36% in 2022). Mentions decreased in the subcategories “sport” (14% in 2021 vs. 12.1% in 2022) and “relaxation” (3.7% in 2021 vs. 1.9% in 2022), while the other subcategories remained stable (“listening to music” with 11.2% vs. 12.1%, “going for a walk” with 4.2% vs. 3.3%, “hobbies” with 3.7% in both 2021 and 2022, “reading” with 2.3% vs. 2.8%).

#### 3.2.3 Distraction

The main category “distraction” was more frequently mentioned as a source of support by students in spring 2022 (16.8% in 2021 vs. 20.1%). While the subcategories “gaming” (4.7% in 2021 vs. 5.1% in 2022) and “surfing the internet” (2.3% vs. 1.4%) remained stable, less respondents mentioned watching TV (3.7% vs. 1.9%). Shopping as a source of support was not an issue in 2022. Instead 1.9% of respondents named “partying” as a resource. The category that saw the largest increase in mentions was “distraction (general category)” (2.3% in 2021 vs. 9.8% in 2022). These mentions were brief and could not be attributed to any other subcategories.

#### 3.2.4 Escapism

The main category “escape” describes a number of avoidance strategies to deal with the distress. It was mentioned more frequently in 2022 than in 2021 (10.7% in 2021 vs. 20.1% in 2022). At both points in time, there was an equal frequency of mentions of the subcategories “eating” (2.8% vs. 2.3%) and “substances” (2.8% vs. 2.3%). The percentage of students who reported that sleeping helped them cope with their burdens increased from 5.6% in 2021 to 8.4% in 2022. In addition, a new subcategory was developed in 2022, which summarizes young people's statements describing the suppression or denial of problems. For example, respondent 210 described, “*The suppression of thoughts or considerations relating to my problems*”, and respondent 642 stated, “*To ignore the submission of homework and not do homework*.”. Non-compliant behavior as a resource was also added as a subcategory in 2022 (1.4%).

#### 3.2.5 Attitudes and abilities

When comparing the data from 2021 and 2022, only slight changes were observed in the category “attitudes and abilities” (18.2% in 2021 vs. 15% in 2022). The use of “mental abilities”, such as attention and breathing exercises, or positive thinking to cope with problems was less frequently mentioned in 2022 (14% in 2021 vs. to 8.9% in 2022). Additionally, responses were not as diverse or elaborated as they were in 2021. Keeping busy and making plans was found helpful by 2.3% of respondents in 2021 and 1.4% in 2022 (subcategory “structure”); the number of respondents who said that expressing their feelings, for example by crying, was helpful in reducing tension remained the same (subcategory “abreact”). Additionally, in 2022, “studying” was added as a category, with 2.8% of mentions.

#### 3.2.6 Further sources of support

In 2022, a new subcategory was created that did not exist in 2021. “Time out” was reported by 7.5% respondents and summarizes responses addressing the use of leisure time, school breaks and weekends as a source of support. For example, respondent 350 cited, “*Time for myself, when I don't have to think about anything else*.”

Other sources of support included “creativity” (4.7% in 2021 vs. 3.3% in 2022), the use of “professional help” (3.3% vs. 2.8%), “faith” (1.4% vs. 0,9%), “school” (1.4% vs. 0.9%), and “pets” (2.3% vs. 0.5%). In 2021, 1.4% of respondents reported not knowing what provided them with support, compared to 0.5% in 2022. 1.4% respectively 2.3% of respondents stated that they didn‘t need support. A more distinct change is evident in the category “nothing”. In 2021, only 3.7% of young people stated that nothing would provide them with support, whereas in 2022, this figure rose to 6.5%.

## 4 Discussion

The objective of our study was to investigate the burdens and resources experienced by Austrian school students after 2 years of the pandemic and compare them to those reported in 2021. A qualitative content analysis was conducted on the responses of 214 young people to two open-ended questions during an online survey in April and May 2022. The results of the analysis were compared with those of a similar study carried out by some of the authors a year earlier (35).

The results reflect the evolving pandemic situation. In 2022, government measures to combat the spread of the virus, such as contact restrictions, travel restrictions, and restrictions on social life were lifted. As a result, pandemic-related concerns, which were the second most common category of burdens in 2021, were no longer mentioned in 2022. The fear of missing out on important live events was not an issue any more for young people in 2022 and concerns about the further development of the pandemic were also no longer relevant in 2022. Since schools resumed face-to-face teaching in February 2022, the burdens of distance learning and changing school routines that were relevant to students in 2021 were no longer present in April and May 2022.

However, the results also show that despite the relaxation of restrictions and a mostly normal school routine, stress at school, i.e. struggles with demands for performance, difficulties in specific subjects and troubles handling the workload of assignments, increased considerably from 43.5% in 2021 to 64.0% in 2022. One possible reason for this increase could be that the 2022 survey was conducted closer to the end of the semester, when school leaving exams and other assessments take place. However, since the proportion of students expressing concern about the upcoming school leaving examination remained stable (7.9% in 2021 vs. 8.9% in 2022), this explanation is not supported. Rather, our findings suggest that at the time of our survey, when regular classes resumed, the demands on students increased. They were faced with the challenge of catching up on missed education after an extended period of alternative teaching models during the pandemic, such as distance learning or hybrid instruction. They were required to take extra exams or prepare for assessments to compensate for learning setbacks. This resulted in increased workloads and stress levels, as has been demonstrated in other studies (21, 30, 33, 34, 58–65). According to Widnall et al. (30), during the pandemic, a gap emerged between students who were able to maintain their motivation and learn independently and those who relied more on in-person lessons. The latter group experienced lasting learning disadvantages as a result of distance learning.

Based on our findings, we recommend implementing additional school-based support and tutoring, particularly for students who experienced setbacks in their academic progress during the pandemic. Previous research has provided evidence that some students were more affected by school-related stress than others. For example, Holtgrewe et al.'s findings showed that insecurity about failing at school was a concern, especially for female students and those who perceived themselves as poor students (66). The authors of a survey carried out in Switzerland in 2020 stressed that students from lower socio-economic backgrounds were more challenged by distance learning (67). Postigo-Zegarra et al. (29) attributed academic overload to the group of young people who experienced difficulties adapting to the pandemic situation. These youth not only suffered from the academic burden but also experienced a sense of loss of routines, social contact, autonomy, freedom, and security. The cited studies, along with our findings, suggest that approaches to supporting young people in finding their way back into the school routine and catching up on missed learning content must take into account different contexts and vulnerabilities. Careful consideration is required to ensure that interventions are appropriately targeted and meet a wide range of needs. Following Widnall et al. (30), we suggest involving young people in developing interventions to ensure acceptable and appropriate responses.

Our study results further indicate that young people not only required cognitive catch-up but also social catch-up. However, returning to a busy social environment was also accompanied by feelings of stress. In comparison to 2021, young people reported experiencing more friendship problems and concerns related to bullying in 2022. While missing out on something was no longer an issue in 2022 and partying was added to the list of resources, respondents commented on a lack of time to fit in all their agendas and a need for more personal time. Respondents in Widnall et al.'s study (30) also emphasized the importance of downtime away from lessons and formal learning, as well as the need for some alone time when readjusting to the familiar patterns of traditional classroom settings, fixed schedules, social interactions, and more structured environments after periods of distance learning.

In 2022, our survey revealed a nuanced reality for young individuals. While some resumed normal activities seamlessly, others grappled with profound existential uncertainties, hinting at struggles beyond daily challenges. Andersen et al.'s existential theory (68) emphasized the pandemic's critical impact on young people's wellbeing, noting a shift away from a performance-oriented mindset toward self-reflection and connection with self-transcending aspects. Relationships, seen as entities larger than oneself, crucially influence how young individuals navigate existential challenges, impacting their journey of self-discovery. The survey transcends traditional mental health measures, revealing a struggle beyond pandemic-related challenges. Exploring identity crises, the quest for meaning, and implications for social relationships deepens our understanding of this existential uncertainty, shedding light on a profound void some young people face. In conclusion, the findings highlight multifaceted existential uncertainty involving identity crises, a search for meaning, and profound implications for social relationships. Delving into these aspects not only enhances understanding but also guides targeted interventions addressing immediate challenges and underlying existential uncertainties. Aligning with Meier's perspective (69), our findings stress the urgency for future studies to explore young people's “existential suffering”, essential for informed interventions addressing the profound uncertainties embedded in their inner lives.

Our study also showed that there was an increase in the mention of specific mental health conditions, such as depression and anxiety. This suggests that respondents improved their health literacy and were better able to identify their emotional state with a certain diagnosis. However, a closer examination of the study's quantitative results supports the interpretation that mental health problems were more prevalent in the 2022 study compared to 2021 (43). The prevalence of clinically relevant symptoms of depression peaked at 73% among girls and 44% among boys (PHQ-9 score ≥ 11). Symptoms of anxiety were prevalent among both girls (57%) and boys (35%) (GAD-7 score ≥ 11). Additionally, the majority of girls (95%) and boys (81%) reported experiencing at least moderate stress (PSS-10 score ≥ 14). Focusing on the young people who reported significant levels of psychological distress in our study, further research should investigate the composition of this vulnerable group, the underlying reasons for their distress and how psycho-social services can best respond to their needs.

In terms of young people's responses to potential sources of support, the results of our study are similar to those of other studies (23, 31, 70, 71). Spending time and talking to friends, family support, recreational activities as well as distraction helped young people to cope with their problems. In addition, it is both noteworthy and worrying that almost one in five respondents in our study reported resorting to escapism as a coping strategy. In particular, young people mentioned an increase in behaviors such as prolonged sleeping and strategies to avoid dealing with their problems. Furthermore, in 2022, more young people reported not knowing what would help them cope with their problems than in 2021. We believe that there is a need for more low-threshold support for young people, both in school and in the private environment, to encourage them to identify problems, talk about them and seek professional help if necessary. Our analysis shows that the percentage of students seeking professional help was very low at both survey dates. Despite the numerous psycho-social initiatives launched during the pandemic in Austria, one would have expected this number to rise at least slightly between 2021 and 2022. Clearly, this is an indication of the need for further initiatives.

This paper investigated the perceived burdens and resources of young people after 2 years of the pandemic. Many COVID-19 restrictions had been lifted at the time of the survey, which is also reflected in the data. Respondents only referred to the pandemic in very few text passages (specifically “family, relationship and interpersonal problems”, as well as “physical health stresses”). Nonetheless, we argue that the pandemic's disruptions continue to have an impact even 2 years after its onset and the lifting of restrictions. Schmitt has highlighted how the COVID-19 pandemic has amplified social problems and inequalities (72). Social, economic, and political disparities and disruptions in society have become more exacerbated and vulnerabilities of people in difficult life situations have increased (72). We need to keep these dynamics in mind, when drafting strategies to counteract their effects.

### 4.1 Implications

Our study showed that even as the pandemic waned, young people's self-reported burden remained high, and in some areas increased. Adolescents' worries and anxieties require careful attention. We suggest a multi-pronged approach: firstly, it is imperative to recognize that many students need tangible support to cope with their academic demands. This requires teaching staff that is sensitive to students' needs and sufficient in number, as well as the provision of ample opportunities for school-based support, such as tutoring or study groups. Secondly, there is a need to ensure that low-threshold psychosocial support is accessible for students, both within the school environment and in the wider environment in which adolescents live. It is also important to ensure that parents or guardians understand the challenges young people face and support their children in coping. This is essential to counteract the stressful and potentially traumatic circumstances they have experienced during the pandemic. Looking to the future, a major effort is needed to assess and cultivate effective teaching methodologies for potential crisis scenarios. There is an urgent need to equip policymakers with the knowledge and strategies necessary to navigate the education sector through future crises.

### 4.2 Limitations

The current study needs to be interpreted within the context of certain limitations. Firstly, the study employed an online survey format where participants were required to provide written responses rather than conducting face-to-face interviews, which could have allowed for more contextually embedded answers. This, for instance, prevented us from gaining a deeper understanding of the factors leading to students' distress. Secondly, the study did not utilize a standardized scale to measure students' concerns and resources; instead, an open-ended question format was employed for exploration. Frequencies of coded categories were calculated to emphasize their significance. Asking respondents to write about their most important current concern after completing a questionnaire focusing on mental health (symptoms) may have led respondents to use language that pathologies normal experiences. Thirdly, it's important to acknowledge that this study involves a comparison of two successive cross-sectional surveys. Longitudinal studies, which track the same individuals over time, could potentially offer a more precise understanding of individual-level changes over time, taking into account inherent variability even in similarly collected samples and potential influences on comparability arising from self-reports. Fourthly, in order to compare the 2021 and 2022 studies, the values of the subcategories were summed up in the respective main category. Methodologically, it would have been sounder to count each respondent only once at the level of the main categories, even if the respondent made statements that were assigned to multiple subcategories. However, this was not possible due to the structure of the 2021 data set. Another shortcoming is the use of convenience sampling, which cannot rule out a self-selection bias toward students with greater mental health problems. Finally, our study was not case-based, thus preventing any comparison of the young people's burdens and resources.

## 5 Conclusions

Our study illustrated that the end of pandemic-related measures did not lead to a decrease in young people's concerns; rather, these concerns manifested and even intensified across various domains. The academic domain emerged as a focal point, indicating that respondents found it challenging to transition back to a normal school routine and catch up on missed learning content. The study found that social contacts and leisure activities can help individuals cope with burdens. However, some respondents found it challenging to balance leisure activities, social contacts, and school tasks when returning to normal life. They desired more time off and resorted to coping strategies such as sleeping more or avoiding problems. Based on our findings, we recommend enhancing low threshold support for young people, including both school-based and private options.

## Data availability statement

The raw data supporting the conclusions of this article will be made available by the authors, without undue reservation.

## Ethics statement

The studies involving humans were approved by Ethics Committee of the University for Continuing Education Krems. The studies were conducted in accordance with the local legislation and institutional requirements. The participants provided their written informed consent to participate in this study.

## Author contributions

MS: Visualization, Writing – original draft. TK: Visualization, Writing – original draft. KH: Formal analysis, Writing – review & editing. RD: Formal analysis, Writing – review & editing. VW: Writing – review & editing. CP: Conceptualization, Writing – review & editing. TP: Writing – review & editing. EH: Conceptualization, Data curation, Formal analysis, Investigation, Methodology, Project administration, Supervision, Writing – review & editing. AJ: Formal analysis, Methodology, Supervision, Writing – review & editing.
