# Supplementary material for: School students' burdens and resources after 2 years of COVID-19 in Austria: a qualitative study using content analysis
Source: Front Public Health. 2024 Feb 9;12:1327999. doi: 10.3389/fpubh.2024.1327999 (PMC10884163; doi:10.3389/fpubh.2024.1327999)
Supplement: Supplementary file 1 [file Table_1.docx]

**Supplementary Table 1.** Category system that emerged from the data for Question 1: “What is currently causing you the most concern?” from the 2021 study.

| **MAIN CATEGORY** | **SUBCATEGORY** |
| --- | --- |
| **School-related concerns** | Stress at school |
|  | School organization |
|  | Graduation |
|  | Teachers |
| **Restrictions** | Restrictions of public life/home confinement |
|  | Lack of activities with friends |
|  | Lack of social contacts |
|  | Lack of exercise |
|  | Travel restrictions |
|  | Quarantine |
| **Self-related concerns** | Concerns about the future |
|  | Negative thoughts and emotions |
|  | Lack of drive |
|  | Missing out on something |
|  | Mental health stresses |
|  | Physical health stresses |
|  | Loneliness and social isolation |
|  | Body-related worries |
| **Family, relationship and interpersonal problems** | Family problems |
|  | Relationship troubles |
|  | Relational problems with others |
|  | Worrying about others |
| **The pandemic** | General statements |
|  | Uncertainty of the future |
| **Current societal development** |  |
| **Other concerns** |  |
| **Consumption** | Alcohol |
|  | Drugs |
|  | Mobile phone/laptop usage |
|  | Sex |
| **No stressors** |  |

**Supplementary Table 2.** Category system that emerged from the data for Question 2: “What is currently providing you with the most support?”

| **MAIN CATEGORY** | **SUBCATEGORY** |
| --- | --- |
| **Social contacts** | Friends |
|  | Family |
|  | Partner |
|  | Talking to someone |
|  | Classmates |
|  | Best friend |
|  | Other people |
| **Recreational activities** | Sports |
|  | Listening to music |
|  | Going for a walk |
|  | Hobbies (general category) |
|  | Relaxation |
|  | Reading |
| **Attitudes and abilities** | Mental abilities |
|  | Structure |
|  | Abreact |
| **Distraction** | Gaming |
|  | Television |
|  | Social Media Activities |
|  | Surfing the internet |
|  | Distraction (general category) |
|  | Shopping |
| **Escape** | Sleeping |
|  | Eating |
|  | Substances |
| **Creativity** | Writing/Drawing |
|  | Making music |
| **Professional help** |  |
| **Pets** |  |
| **Faith** |  |
| **School as a resource** |  |
| **I don`t know** |  |
| **Nothing** |  |
| **I don`t need help** |  |

**Supplementary Table 3.** Category system that emerged from the data for Question 1: “What is currently causing you the most concern?”

|  | N | % |
| --- | --- | --- |
| **School‐related concerns** | **161** | **75.2** |
| Stress at school | 137 | 64.0 |
| Graduation | 19 | 8.9 |
| Teachers | 4 | 1.9 |
| School organization | 1 | 0.5 |
| **Self‐related concerns** | **69** | **32.2** |
| Mental health stresses | 18 | 8.4 |
| Negative thoughts and emotions | 12 | 5.6 |
| Lack of time | 11 | 5.1 |
| Concerns about the future | 10 | 4.7 |
| Lack of drive | 5 | 2.3 |
| Loneliness and social isolation | 4 | 1.9 |
| Physical health stresses | 3 | 1.4 |
| Mobbing | 2 | 0.9 |
| Body‐related worries | 2 | 0.9 |
| Making decisions | 2 | 0.9 |
| **Family, relationship and interpersonal problems** | **37** | **17.3** |
| Relational problems with others | 15 | 7.0 |
| Family problems | 13 | 6.1 |
| Relationship troubles | 8 | 3.7 |
| Worrying about others | 1 | 0.5 |
|  |  |  |
| **Other concerns** | **21** | **9.8** |
|  |  |  |
| **Current societal development** | **10** | **4.7** |
|  |  |  |
| **No stressors** | **9** | **4.2** |
|  |  |  |
| **The pandemic** | **6** | **2.8** |
|  |  |  |
| **Consumption** | **2** | **0.9** |
|  |  |  |
| **Restrictions** | **1** | **0.5** |

The percentages of the main categories correspond to the sum of the percentages in the individual subcategories. It may be that a respondent reported experiences in several subcategories (e.g., family problems and relationship troubles) within one main category (e.g., family, relationship and interpersonal problems). Therefore, some participants might appear more than once per main category.

**Supplementary Table 4.** Category system that emerged from the data for Question 2: “What is currently providing you with the most support?”

|  | **N** | **%** |
| --- | --- | --- |
| **Social contacts** | **136** | **63.6** |
| Friends | 68 | 31.8 |
| Family | 21 | 9.8 |
| Talking to someone | 20 | 9.3 |
| Partner | 16 | 7.5 |
| Other people | 5 | 2.3 |
| Classmates | 3 | 1.4 |
| Best friend | 3 | 1.4 |
| **Recreational activities** | **77** | **36.0** |
| Sports | 26 | 12.1 |
| Listening to music | 26 | 12.1 |
| Hobbies (general category) | 8 | 3.7 |
| Going for a walk or drive | 7 | 3.3 |
| Reading | 6 | 2.8 |
| Relaxation | 4 | 1.9 |
|  |  |  |
| **Escape** | **43** | **20.1** |
| Sleeping | 18 | 8.4 |
| Repression | 11 | 5.1 |
| Substances | 6 | 2.8 |
| Eating | 5 | 2.3 |
| Non-compliant behavior | 3 | 1.4 |
| **Distraction** | **43** | **20.1** |
| Distraction (general category) | 21 | 9.8 |
| Gaming | 11 | 5.1 |
| Television | 4 | 1.9 |
| Partying | 4 | 1.9 |
| Surfing the internet | 3 | 1.4 |
|  |  |  |
| **Attitudes and abilities** | **32** | **15** |
| Mental abilities | 19 | 8.9 |
| Studying | 6 | 2.8 |
| Abreact | 4 | 1.9 |
| Structure | 3 | 1.4 |
|  |  |  |
| **Time out** | **16** | **7.5** |
|  |  |  |
| **Nothing** | **14** | **6.5** |
|  |  |  |
| **Creativity** | **7** | **3.3** |
|  |  |  |
| **Professional help** | **6** | **2.8** |
|  |  |  |
| **I don`t need help** | **5** | **2.3** |
|  |  |  |
| **School** | **2** | **0.9** |
|  |  |  |
| **Faith** | **2** | **0.9** |
|  |  |  |
| **Pets** | **1** | **0.5** |
|  |  |  |
| **I don`t know** | **1** | **0.5** |

The percentages of the main categories correspond to the sum of the percentages in the individual subcategories. It may be that a respondent reported experiences in several subcategories (e.g., friends, family) within one main category (e.g., social contacts). Therefore, some participants might appear more than once per main category.
